# Prevalence and determinants of contraceptive discontinuation among reproductive age women: analysis of Tanzania demographic health survey

**DOI:** 10.3389/fgwh.2025.1393020

**Published:** 2025-04-11

**Authors:** Bezawit Melak Fente, Angwach Abrham Asnake, Yohannes Mekuria Negussie, Meklit Melaku Bezie, Zufan Alamrie Asmare, Hiwot Altaye Asebe, Beminate Lemma Seifu

**Affiliations:** ^1^ Department of General Midwifery, School of Midwifery, College of Medicine & Health Sciences, University of Gondar, Gondar, Ethiopia; ^2^ Department of Epidemiology and Biostatistics, School of Public Health, College of Medicine and Health Sciences, Wolaita Sodo University, Wolaita Sodo, Ethiopia; ^3^ Department of Medicine, Adama General Hospital and Medical College, Adama, Ethiopia; ^4^ Department of Public Health Officer, Institute of Public Health, College of Medicine and Health Sciences, University of Gondar, Gondar, Ethiopia; ^5^ Department of Ophthalmology, School of Medicine and Health Science, Debre Tabor University, Debre Tabor, Ethiopia; ^6^ Department of Public Health, College of Medicine and Health Sciences, Samara University, Samara, Ethiopia

**Keywords:** prevalence, contraceptive, determinants, discontinuation, women, TDHS 2022, Tanzania

## Abstract

**Background:**

Abortions performed unsafely, unintended births, and missed and unwanted pregnancies are linked to discontinuation of contraception for reasons other than wanting to become pregnant, and these situations raise the risk of maternal morbidity and death. However, a study on the determination of factors contributing to contraceptive discontinuation in Tanzania is limited. Therefore, we aimed to investigate the prevalence and determinants of contraceptive discontinuation among reproductive-age women in Tanzania using recent Tanzania Demographic Health Surveys.

**Method:**

A cross-sectional study was conducted using secondary data analysis from of 2022 Tanzania Demographic Health Survey (DHS). A total weighted sample of 6,467 reproductive-age women were included. To account for the clustering effects of DHS data and the binary nature of the outcome variable, a multilevel binary logistic regression model was applied. An adjusted odds ratio with a 95% confidence interval was reported to declare the statistical significance. In addition, the model that had the lowest deviance was the one that best fit the data.

**Result:**

The prevalence of discontinuation for all contraceptive methods among reproductive-age women was 34% (95% CI: 31.3%, 34.7%). Women who age group of 20–29 years (AOR = 4.45, 95% CI: 1.11, 17.78), women with no formal education (AOR = 1.94, 95% CI: 1.71, 2.93), women having no children (AOR = 4.25, 95% CI: 3.47, 8.06) women who want another child (AOR = 1.89, 95% CI: 1.76, 3.46), distance to the health facility as a big problem (AOR = 2.43, 95% CI: 1.38, 4.26), rural residence (AOR = 1.67, 95% CI: 1.48, 3.23) Were factors strongly associated with contraceptives discontinuation.

**Conclusion:**

Among women of reproductive age, the withdrawal of contraception was 34% and it was determined by several factors. Providing a variety of contraceptive techniques and spreading knowledge about family planning are the primary goals of contraceptive counseling. It is also suggested by prospective investigators to use primary data to address independent factors that were missing.

## Introduction

Because family planning has a direct and indirect impact on maternal mortality, it is a vital component of safe motherhood programs (1). Effective contraceptive use may save up to 44% of maternal fatalities, according to studies and it is commonly known that unintended pregnancies influence maternal mortality and morbidity (2). Contraceptive discontinuation reduces the contraceptive utilization coverage and family planning program effectiveness, as well as contributes to undesired fertility (3). Reducing the number of methods of contraception that a client must choose from, along with giving them access to additional information, technical expertise, interpersonal relationships, follow-up and continuity mechanisms, and the right constellation of services, can all help to improve the quality of their overall care (4).

In some low- and middle-income countries (LMIC) rates of contraceptive discontinuation remain high and increasing, even among women who want to avoid pregnancy (5). Results from a 2012 meta-analysis of 60 DHS surveys, which combined all contraceptive methods across 25 LMICs showed that, of women who started using a contraceptive method, on average 38% stopped within the first year, and two-thirds (68%) stopped before 2 years (6). More than half of the women who stopped contraceptive use, had experienced contraceptive discontinuation or had method-related problems, and were still in need of an effective method to prevent unintended pregnancy (7, 8).

Contraceptive discontinuation is a worldwide public health concern in countries with low and middle incomes (9). An estimated 74 million women worldwide become pregnant each year without any prior planning (10). Unwanted pregnancies result from a variety of circumstances, such as inconsistent and improper use of methods of contraception, failure of contraceptives, termination of contraception, and non-use of contraception (6, 11). Discontinuing contraceptive use is characterized as beginning use and then ceasing for whatever reason while being at risk of an unplanned pregnancy (12).

Enhancing service delivery and user uptake of contraception requires an analysis of the causes of discontinuation of contraceptives. For instance, it was discovered that two-thirds of sexually active women who did not wish to become pregnant discontinued due to fear of side effects, health issues, and underestimating the chance of conception (13, 14). Discontinuation because of side effects, may suggest the need for improved counseling and communication (15). Other reported reasons for discontinuation include accessibility to contraceptives, cost of services, opposition and religious beliefs as well as misunderstanding of how to use the contraceptives (12, 16).

Numerous variables may influence the decision to continue or stop using contraceptives, like as; the age of the woman, number of living children (parity), desired number of children, having television and radios, decision-maker to use contraceptives, husband/partner support, perceived benefit to the FP, perceived contraceptive harm, duration of contraceptive use, counseling on FP and contraceptive side effects, experience of side effects, access and availability different type of contraceptive (3, 17–29).

There have been studies into the identification of factors associated with contraceptive use in Tanzania, but as to our search of the literature, there is limited study on the prevalence and associated factors of contraceptive discontinuation among reproductive-age women in Tanzania (30). The findings of this study can provide direction for policymakers, programmers, healthcare providers, and other stakeholders about the impact of contraceptive discontinuations on morbidity and mortality. This study's findings strongly advocated for women's empowerment, partner involvement in the decision-making process around contraception use, and easy access to high-quality counseling regarding the possible negative consequences and alternative birth control methods. To meet the SDGs that aim to reduce maternal mortality of children, policymakers, programmers, and other stak eholders will be better equipped to support family planning and other health intervention initiatives. Therefore, this study investigated the prevalence of contraceptive discontinuation and associated factors among reproductive-age women in Tanzania.

## Methods and materials

### Data source and sampling procedure

This study was based on the Demographic and Health Survey (DHS) of Tanzania. The Tanzania Demographic and Health Survey (TDHS) is conducted every five years to generate updated health and health-related indicators. The data were derived from the measure DHS program and detailed information about the surveys can be found in each country's DHS reports. The sample design for the 2022 TDHS was carried out in two stages. In the first stage, 629 Enumeration Areas (EAs) were recruited; 211 were from urban areas and 418 were from rural areas. Second stage, 26 households were selected. All women aged 15–49 who were either usual residents or visitors in the household on the night before the survey were eligible to be interviewed in the 2022 TDHS. The sampling frame excluded institutional populations, such as persons in hospitals, hotels, barracks, camps, hostels, and prisons. There are different datasets in DHS, and for this study, we used the Individual Record (IR) file. The dependent and independent variables were extracted from the IR dataset, based on the literature. The final weighted sample size was 6,467.

### Study variables

#### Dependent variable

The outcome variable for this study was modern contraceptive discontinuation (yes/no) among reproductive-age women (women aged 15–49 years), which is measured as the percentage of reproductive-age women who used a method of contraception in the last 12 months before the survey but were not using a method at the time of data collection (19, 25, 29).

#### Independent variables

Individual level variables respondent's age, occupation, education, marital status, husband's fertility desire, women's fertility preference, decision maker on contraceptive use, number of living children, history of visiting health facility, heard about family planning, household head sex, wealth status, and media exposure on family planning. All of those variables were selected based on the literature review from previous studies. We have categorized respondent age based on the previous literature but other variables were categorized by the DHS data initially.

Community-level variables in addition to place of residence, to assess affect group-level variation which is the community-level poverty and education level of the community were included. Community-level variables are calculated based on the number of clusters included in the study. The community level variable and the clustered are run together in the Stata. The value of those two variables' proportion was calculated in an Excel spreadsheet to get the community-level variable, and then it was dichotomized based on the normality. Finally, mean and median were used for normal and skewed distribution respectively.

### Statistical analyses

Descriptive analysis was performed using frequency and percentage distributions to examine the characteristics of respondents and contraceptive discontinuation. This was followed by bivariate multilevel logistic regression to select variables that had a significant association with contraceptive discontinuation at a *p*-value less than 0.05. A multicollinearity test was performed using variance inflation factor (VIF) for all statistically significant variables at the bivariate multilevel logistic regression. Using the Multilevel Logistic Regression (MLLR) method, we created four different models to assess whether the individual/household and community-level factors had significant associations with the outcome variable (contraceptive discontinuation). The first model was a null model (Model 0), which had no explanatory variables and it showed variance in contraceptive discontinuation. The second model (Model I) comprised individual/household-level factors and the third model (Model II) comprised community-level factors. The last model, (Model III), was the complete model that included factors at both the individual/household and community levels.

All four MLLR models included fixed and random effects (31, 32). The fixed-effect model showed the association between the explanatory variables and the outcome variable, and the random effects signified the measure of variation in the outcome variable based on the primary sampling unit, which was measured by Intra-Cluster Correlation (ICC) (33). The model ft. was assessed using the Akaike's Information Criterion (AIC) (34). We used the “melogit” command to run the MLLR models. The analyses were performed using Stata version-14 software (Stata Corp, College Station, Texas, USA). We also followed the guidelines for Strengthening Observational studies in Epidemiology (STROBE) (35).

## Result

### Background characteristics of respondents

A total of 6,467 women 5 years preceding the survey were included in this study. About 2,208(36.21%)women were found in the age groups of 20–29 years followed by the age groups of 30–39 years 1,663 (25.72%). Most women (61.12%) lived in rural areas, and nearly half of women (46%) had attained primary education. The majority of women (36.9%) had jobs, more than half of women (60%) were married, and (56.37%) of women heard about family planning (Table 1).

**Table 1:** Socio-demographic, reproductive, and obstetric characteristics of reproductive-age women in Tanzania, TDHS 2022 (weighted).

Variable	Category	Frequency	Percent (%)
Age	>20	1,332	20.60
	20–29	2,208	36.21
	30–39	1,663	25.72
	40–49	1,263	17.52
Occupation	Not working	2,386	36.90
	Working	4,080	63.09
Marital status	Married	3,880	60.0
	Never married/divorced/widowed/separated	2,587	40.0
Education	No education	1,012	15.65
	Primary	2,975	46.0
	Secondary	2,291	35.42.
	Higher	158	2.44
Residence	Urban	2,514	38.88
	Rural	3,953	61.12
Sex household	Male	4,527	70.00
	Female	1,940	30.00
Wealth status	Poorest	1,028	15.89
	Poorer	1,059	16.38
	Middle	1,299	20.08
	Richer	1,432	22.15
	Richest	1,649	25.51
Heard about family planning	No	2,822	43.63
	Yes	3,645	56.37
Own mobile	No	2,532	39.15
	Yes	3,935	60.85
Number of children	None	1,716	26.5
	1–2	1,888	29.20
	3–4	1,560	24.13
	5 more	1,302	20.13
Visit health facility last 12 months	No	2,781	43.00
	Yes	3,686	57.000
Respondent opinion compared to husband's on use of contraception	More important	867	13.41
	Equally important	5,338	82.54
	Less important	259	4.04
Decision maker for using contraception	Respondent	1,190	18.41
	Husband/partner	545	8.53
	Joint decision	4,732	73.06
Husband's desire for children	Both want same	2,340	38.19
	Husband wants more	1,511	24.68
	Husband wants fewer	398	7.16
	Don’t know	2,009	31.07
Fertility preference	Have another	3,907	60.42
	No more	1,608	24.86
	Undecided	1,015	15.70
Distance health facility	Big problem	1,638	25.34
	Not a big problem	4,828	74.66
History of terminated pregnancy	No	5,357	82.83
	Yes	1,110	17.17
Community educational level	Low	3,266	50.05
	High	3,230	49.95
Community level wealth	Rich	324	50.11
	Poor	13,226	4,989

### Prevalence of contraceptive discontinuation

The prevalence of contraceptive discontinuation among women of reproductive age in Tanzania was 34% with a 95% CI (31.3%, 37.6%). The most common reasons given for discontinuing the contraceptive method were the desire to become pregnant, side effect concerns, wanting more of an effective method, husband/partner disapproval, unexpected conception, and other concerns. More than two-thirds (36.4%) of women who discontinued contraceptive use desired to become pregnant, (12.7%%) of discontinuations were due to changes in menstrual bleeding (19.3%) were concerned about the other side effects, and wanted a more effective method (Table 2).

**Table 2:** Reasons for discontinuation of contraceptives.

Reason for the discontinuation	Frequency	Percent
Wanted to become pregnant	1,240	36.4
Side effects	639	19.3
Changes in menstrual bleeding	387	12.7
Wanted a more effective method	265	8.2
Other	217	5.9
Contraceptive failure	190	4.8
Infrequent sex, husband away	130	3.7
Access, availability	81	2.8
Husband disapproved	79	1.9
Inconvenient to use	54	1.5
Marital dissolution	45	0.8
Difficult pregnancy, menopause	19	0.5
Don’t know	19	0.5
Cost	16	0.5
Fatalistic	11	0.4

### Factors associated with contraceptive discontinuation

In the multivariable mixed effect binary logistic regression model, women's age, women's education status, women need more children (fertility preference), and distance from health facility significant individual factors while the place of residence was found to be a statistically significant factor from community-level factors of contraceptive discontinuation among Tanzanian reproductive age women.

Women in who age group of 20–29 years are 4.45 times more likely to discontinue contraceptives (AOR = 4.45 CI: 1.11, 17.78). Women with no formal education were 1.94 times more likely to discontinue contraceptives (AOR = 1.94, 95% CI: 1.71, 2.93). Women having no children (AOR = 4.25, 95% CI: 3.47, 8.06) were 4.25 times more likely to discontinue contraceptives compared to women with five or more children. In addition, women who reported that wanted another child were nearly two times more likely to discontinue contraception (AOR = 1.89, 95% CI: 1.76, 3.46) than those not want more children. The odds of discontinuing contraceptives among women who perceived the distance to the health facility as a big problem was increased by 2.43 times (AOR = 2.43, 95% CI: 1.38, 4.26) than women who perceived the distance to the health facility as not a big problem. Women with rural residences were 1.67 times more likely to discontinue contraceptives (AOR = 1.67, 95% CI: 1.48, 3.23) compared to those women with urban residences (Table 3).

**Table 3:** Multilevel analysis of factors associated with contraceptive discontinuation and random effect among reproductive-age women in Tanzania, 2022 (*N* = 6,467).

Variable	Null model	Model I	Model II	Model III
Age				
15–19		2.93 (2.18, 3.93)		3.91 (0.89, 17.22)
20–29		3.83 (2.90, 5.06)		**4.45** (**1.11, 17.78)**
30–39		1.05 (0.76, 1.44)		1.71 (0.14, 3.51)
40–49		1		1
Education				
No education		1.14 (1.00, 1.31)		**1.94** (**1.71, 2.93)**
Primary		1.13 (0.96, 1.34)		1.36 (0.55, 3.36)
Secondary		1.10 (0.73, 1.66)		3.21 (0.53, 19.23)
Higher		1		1
Visit health facility				
Yes		1		1
No		1.65 (1.49, 1.82)		1.61 (0.96, 2.70)
Occupation				
Not working		1		1
Working		1.43 (1.29, 1.59)		1.00 (0.57, 1.76)
Marital status				
Married		1		1
Never married/divorced/widowed/separated		2.74 (1.74, 4.32)		0.68 (0.11, 4.19)
Number children				
None		8.67 (6.79, 11.06)		**4.25** (**3.47, 8.06)**
1–2		1.48 (1.40, 1.63)		**9.10** (**8.11, 72.28)**
3–4		1.37 (0.92, 1.8)		2.56 (0.99, 3.92)
5 and more		1		1
Heard about family planning				
Yes		1		1
No		1.07 (0.97, 1.19)		1.08 (0.63, 1.85)
Decision maker				
Respondent		1		1
Husband/partner		0.56 (0.46, 0.68)		0.55 (0.21, 1.48)
Joint decision		1.05 (0.93, 1.18)		1.04 (0.57, 1.89)
Husband desires to have another child				
No		1		1
Yes		4.58 (2.94, 7.13)		2.17 (0.37, 12.55)
Fertility preference				
Need more		2.05 (1.92, 2.20)		**1.89** (**1.76, 3.46)**
Undecided		0.75 (0.65, 0.87)		0.72 (0.28, 1.80)
No more		1		1
Distance to a health facility				
Not a big problem		1		1
Big problem		1.88 (1.78, 1.99)		**2.43** (**1.38, 4.26)**
Wealth status				
Poorest		1		1
Poorer		1.22 (1.03, 1.45)		0.54 (0.25, 1.16)
Middle		1.34 (1.13, 1.60)		0.82 (0.34, 1.97)
Richer		1.34 (1.11, 1.62)		1.35 (0.58, 3.11)
Richest		1.49 (1.21, 1.83)		1.18 (0.41, 3.39)
Own mobile				
Yes		1		1
No		1.24 (1.10, 1.39)		0.71 (0.39, 1.27)
Sex household				
Female		1		1
Male		1.03 (0.91, 1.16)		1.17 (0.64, 2.15)
History of pregnancy termination				
No		1		1
Yes		1.09 (0.97, 1.23)		1.77 (0.87, 3.60)
Residence				
Urban			1	1
Rural			1.75 (1.25, 2.08)	**1.67** (**1.48, 3.23)**
Community wealth				
Poor			1	1
Rich			1.02 (0.66, 1.57)	1.01 (0.60, 1.70)
Community Education				
Low			1	1
High			0.94 (0.61, 1.45)	0.96 (0.57, 1.61)
Log-likelihood	−7,891.5576	−6,438.6616	−364.50877	−251.74829
ICC (%)	12.2	11.1	10.3	10.1
AIC	15,787.12	12,933.32	739.0175	565.4966
BIC	15,802.38	13,147.04	761.2302	703.2153
Deviance	15,783.115	12,877.323	729.0175	503.49658
MOR (95% CI)				
PCV (%)		9.1%	71%	73%

ICC, intra-class correlation coefficient; MOR, median odds ratio.

Bold indicates significantly associated values.

### Random effects (measures of variations) results

The random effect models of the individual/household and community-level factors associated with contraceptive discontinuation are shown in (Table 3). We observed that the values of the AIC and deviance decreased across the models, indicating the best-fitted model was chosen based on the lowest deviance value (503.49658) and AIC (565.966). The ICC in the null model (ICC = 12.2%) showed that the odds of contraceptive discontinuation varied across clusters. The between-cluster variations decreased by 1.1% in the model I, from 12.2% in the null model to 11.1% in the model I. From model I, the ICC decreased again by 2% in model II (ICC = 1.3%) and then declined by 2.1% in the complete model (model III, ICC = 10.1%. These estimates showed that the variations in the likelihood of contraceptive discontinuation can be attributed to the variances in the clustering at the primary sampling units (Table 3).

## Discussion

In this study, we investigated contraceptive discontinuation and its individual/household and community-level factors among women of reproductive age using a recent Tanzanian nationally representative dataset. According to the current study; 34% with 95% CI (31.3%, 37.6%). of Tanzanian women who started an episode of contraceptive use preceding TDHS were discontinued within 12 months. It was consistent with studies reported in Southeast Ethiopia which showed that about 2 of women discontinue contraceptives (28). This finding was higher than the DHS survey conducted from 34 countries, which reported a discontinuation rate of 19% (14), and Kenya's 30.5% in 2014 DHS (26), which reported the prevalence of contraceptive discontinuations, However, this finding is lower than study findings from 60 surveys in 25 countries with a discontinuation rate of 38% (28), analysis of Bangladesh DHS with 38.4% (24), Myanmar with 38% (22), Ethiopia (46.1%) (29), and rural Bangladesh (36%) (36). This variation may result from variations in reproductive health services, such as information, counseling, and education that can improve women's comprehensive knowledge of FP, the accessibility and readily accessible nature of various FP methods, and the development of easy and secure services for users of contraceptives. Furthermore, socio-demographic differences, norms, beliefs, and other cultural differences may be the reason for the discrepancy. The study period, sample size, study population, and sampling technique may be the additional cause.

According to this study, the rate of younger women discontinuing any kind of contraception was higher. This finding is consistent with research from Bangladesh (24), and Ghana (37). Our results are not surprising, as the highest levels of fertility are typically found in younger women. Put in another context, as these women are more likely to have started actively producing children, they are also more likely to have stopped taking contraception if they had previously used it.

In this study, women who lived in a rural residence were 1.75 times more likely to discontinue contraceptives compared with those living in an urban residence. This result is supported by other studies (24, 25). This may be explained by the fact that women in the countryside have limited access to services for family planning and reproductive health information (6). Similarly, women who perceived distance to reach health facilities as a big problem had higher odds of discontinuing contraceptives than those who perceived it as not a big problem. It can be explained that women who had poor healthcare access might not use family planning services.

Women with no formal education were two times more likely to discontinue contraceptives. This is also similar to other findings (3, 24, 25, 37). The reason might be that women with no formal education have been deprived of autonomy to make decisions on reproductive health issues including when to use contraceptives. Women who had no children or fewer than three children were more likely to discontinue contraceptives than their counterparts. This finding is supported by other studies (18, 22, 25, 28). The reason might be that women may need to have children to attain their desired number of children; thus they might prefer to discontinue contraceptives.

Another significant predictor of discontinuing contraceptives was women who want more children This was consistent with previous studies (19). This can be because women stop using contraceptives because they want to have more children. This study can provide great information for the policymakers and for the Tanzanian health facilities to improve the morbidity and mortality related to contraceptive discontinuations.

### Strengths and limitations of the study

One of the study's advantages was that it used data from a large nationwide survey, which gave it sufficient power to identify the real impact of the independent factors. Secondly, in order to obtain accurate estimates and standard errors, the sample weight was applied during the analysis. Furthermore, by examining the appropriate cessation of contraceptives at the household, and community levels, we were able to investigate hierarchical or clustered patterns that might have an impact on results. One weakness of the study is that it was cross-sectional in nature; therefore it was not possible to establish a causal relationship between the identified independent variables and the discontinuation of contraceptives. Because it depends on self-reported data, the DHS is vulnerable to recall bias.

## Conclusion

Contraceptive discontinuation among reproductive-aged women was 34%in Tanzania in this study. Age, educational status, number of children, women's fertility preference, and distance from health facility were significantly associted factors from individual-leve variables while place of residence was significant associated factor from community-level variable with contraceptive discontinuation. Improving family planning awareness through comprehensive reproductive education or counseling could be one of the operational ways to reduce contraceptive discontinuation. Counseling on contraceptives usually focuses on disseminating information on family planning and providing a range of contraceptive methods. Initiation of a contraceptive method has been linked to a lower risk of unplanned pregnancy. Future researchers are also recommended to use advanced or fellow up study to predict the exact cause and effect relationship.

## Data Availability

The original contributions presented in the study are included in the article/Supplementary Materials, further inquiries can be directed to the corresponding author.
